# Is There an Association between Socioeconomic Status and Body Mass Index among Adolescents in Mauritius?

**DOI:** 10.1100/2012/750659

**Published:** 2012-04-19

**Authors:** Waqia Begum Fokeena, Rajesh Jeewon

**Affiliations:** Department of Health Sciences, Faculty of Science, University of Mauritius, Réduit, Mauritius

## Abstract

There are no documented studies on socioeconomic status (SES) and body mass index (BMI) among Mauritian adolescents. This study aimed to determine the relationships between SES and BMI among adolescents with focus on diet quality and physical activity (PA) as mediating factors. Mauritian school adolescents (*n* = 200; 96 males, 104 females) were recruited using multistage sampling. Participants completed a self-reported questionnaire. Height and weight were measured and used to calculate BMI (categorised into underweight, healthy-weight, overweight, obese). Chi-square test, Pearson correlation, and Independent samples *t*-test were used for statistical analysis. A negative association was found between SES and BMI (*χ* ^2^ = 8.15%, *P* < 0.05). Diet quality, time spent in PA at school (*P* = 0.000), but not total PA (*P* = 0.562), were significantly associated with high SES. Poor diet quality and less time spent in PA at school could explain BMI discrepancies between SES groups.

## 1. Introduction

Paediatric obesity, a multifactorial problem, is reaching epidemic levels in developed countries [1]. It is even penetrating the world's poorest countries especially in their urban areas [2]. The fact that about 70% of obese adolescents grow up to become obese adults [3] highlights the urgency to tackle the problem as early as possible. Mauritius, a developing country with upper middle income economy, has around 1.2 million inhabitants of which 10.2% are adolescents [4]. To date, it has been found that 8.4% of Mauritian adolescents are overweight while 7.3% are obese [5]. Studies on the problem of pediatric obesity, however, are scanty in Mauritius.

The increase in prevalence of adolescent obesity is a manifestation of the epidemics of sedentary lifestyle and excessive energy intake [6]. While environmental factors affecting energy intake and expenditure such as diet composition, portion size [7] sedentary, and physical activities [8] are well documented; the role that social and economic environment of a person play in influencing his or her energy intake and expenditure, and hence BMI, is often overlooked. This should not have been the case as cost is reported to be an important factor in food selection [9].

As far as the relationships between socioeconomic status (SES) and body mass index (BMI) are concerned, a consistent and strong negative link has been established in most developed countries [10–15]. The opposite has been found in some (urban India and Ghana) [16, 17] but not all developing countries. For example, in Iran, prevalence of obesity was lower among high-income elderly [18]. Several studies have reported inconsistencies in the SES-BMI relationship [19–21]. For instance in Hong Kong, SES had no significant effect on childhood BMI [19] and in Iran, parental education and income were poor predictors of BMI among adolescent girls [21]. To date, only one unpublished study on the prevalence of obesity among adolescents conducted in Mauritius examined the effect of SES on obesity, and no correlation was found [unpublished]. These findings demonstrate that there is still a loophole regarding the link between SES and BMI.

Another important aspect, found to be a key intermediate in the SES-BMI relationship, is diet quality. Studies in developed countries have demonstrated that a healthy diet is mostly present among high SES individuals, which might account for the negative relationship between SES and BMI reported in several countries [22–26]. For instance, a review paper reported that whole grains, lean meats, fish, low-fat dairy products, and fresh vegetables and fruits are consumed mostly by high income groups while refined grains and added fats are associated with lower SES [27]. The calorie-cost relationship might explain the choice of calorie-dense food by low SES groups in developed countries [28]. However, this is not the case in less developed countries. Among rural adolescents in India, as SES increases, there is a proportional rise in energy intake [29].

Results from previous studies suggest that there are still controversies regarding the link between SES and energy expenditure. While in 1999, it was reported that neither physical activity (PA) nor sedentary behaviours mediate the SES-BMI relationship [15], another study in 2010 found that both may influence this link depending on the methods used to determine PA and sedentary behaviours [30]. Previously, it has also been documented that only sedentary behaviours such as watching television and playing video games influence the relationship between SES and BMI, and not PA [12]. However, most studies have reported a positive relationship between SES and BMI [31–33]. Nevertheless, possible factors which could explain sedentary behaviours and/or lack of PA in youth from low SES backgrounds are a lack of social encouragement due to low parental education [31, 34], poor neighbourhood safety [12, 35], and low accessibility to recreational resources [36, 37]. An additional uninvestigated factor is the time spent in physical activity at school. This would give an indication of the conduciveness of low and high SES schools to increase energy expenditure. Children and adolescents spend a significantly large amount of their time at school, an ideal place to promote PA and decrease sedentary behavior [38].

Given that a complex relationship exists between SES, PA, and diet quality, on weight problems in youngsters, this study was undertaken to understand the relationships between SES and BMI among adolescents, in a Mauritian context. The main objectives were (i) to determine the association between SES and BMI, (ii) to identify any link between SES and diet quality, and its influence on BMI, and (iii) to find whether SES affects PA level (as well as time spent in PA at school), and if this is reflected in participants' BMI.

## 2. Method

### 2.1. Participants

Using a multistage sampling method, a total of 200 participants from both genders, aged between 12 and 15 years, were involved in the study. Age was calculated from date of birth and date on which participants took part in survey. The sample was taken from three fee-paying and three private-aided schools, which consist mainly of high and low SES participants, respectively. Schools were chosen at random from the four educational zones of Mauritius [42]. To further confirm participants' SES, the family affluence scale (FAS) [43] was adapted and used. FAS included four questions: “How many mobile phones are currently being used in your family?,” “In all, how many four-wheeled motor-vehicles does your family possess,” and “Do you have a bedroom of you own which is unshared?”. The scores were grouped into two levels: low SES (from 0 to 2) and high SES (from 3 to 5). Parental consent forms were signed prior to participation. Adolescents who were taking medications which promote weight gain (steroids, oral contraceptives, tricyclic antidepressants) or those following weight-gain/weight-loss therapy did not participate. Adolescents suffering from influenza or had hormonal disorders, which might affect energy balance, were also excluded.

### 2.2. Questionnaire

A 33-item coded questionnaire was used. Demographic data (age, gender, date of birth) and information for the FAS were collected. A food frequency table (FFT), adapted from that of Dynesen et al. [44], was included to assess participants' diet quality. Dietary guidelines for the prevention of noncommunicable diseases for Mauritian adolescents aged from 13 to 18 years [41], American Heart Association dietary guidelines for children and adolescents [40] and United States Department of Agriculture MyPyramid guidelines for kids 2005 [39] were merged to produce a list of 12 dietary guidelines each addressing a particular food group of the FFT. Scores assigned to the frequencies (more than once daily = 4; once daily = 3; once or more per week = 2; once or more per month = 1; rarely/never = 0) were used to compare consumption of the twelve different food items. Depending on participants' consumption frequency scores, it was determined whether dietary guidelines were being followed (1 = dietary guideline followed; 0 = dietary guideline not followed) (Table 1). Physical activity (PA) was calculated in metabolic equivalents (MET)—minute per week—and categorized into low, moderate, and high PA levels, using the validated International Physical Activity Questionnaire-short last 7 days self-administered format [45]. Participants were also asked to report the number of minutes spent on physical activity at school per week.

### 2.3. Market Survey

A market survey was conducted (in both urban and rural areas) on the cost (in Mauritian rupees (Rs); US$ 1 approximately Rs 30) per 100 Cal and cost per 100 g of food items listed in the FFT to determine whether calorie-density [46] and price of food items influenced food choice among the two SES groups. Calorie content of food items was calculated using food composition tables [47].

### 2.4. Anthropometry

Anthropometric measurements were taken using the same instruments in all the schools. Adolescent height and weight were measured using a standard protocol [48]. Height was measured without shoes to the nearest 0.05 m (standard error ±  0.05 m). Weight was measured without shoes in school uniforms (pockets were emptied) to the nearest 0.5 kg (standard error ±  0.5 kg). BMI was calculated to one decimal place and classified into underweight, healthy-weight, overweight, and obese using the age-adjusted standardized BMI percentile distribution cut-off points for children and adolescents developed by the National Center for Health Statistics, Centre for Disease Control and Prevention [49].

### 2.5. Statistical Analysis

All statistical analyses were conducted using SPSS 17.0. Alpha value was adjusted to 0.05. Chi-square test for independence was used to determine the difference between the two SES groups for BMI and PA. Independent sample *t*-test was used to compare groups (low and high SES) for a continuous variable (BMI, scores on each food item, total scores on dietary guidelines met, PA) and Pearson product-moment correlation used to explore the relationship between PA and BMI.

## 3. Results

### 3.1. Demographic Characteristics

Of the 200 participants, 102 (51.0%) were from low SES and 98 (49.0%) from high SES. Roughly equal number of males (48%) and females (52%) were noted for both SES groups (96 males/104 females). The average age of participants was 13.6 years.

### 3.2. Socioeconomic Status and Body Mass Index

There was a significant relationship between SES and body mass index (BMI) among the participants, *χ* ^2^ (1, *n* = 200) = 8.15, *P* = 0.0430. Mean BMI for the low SES group was higher (19.9 ± 4.41) than that of the high SES group (18.5 ± 2.83). The percentage of underweight, overweight, and obese was higher among the low SES participants (Table 2).

### 3.3. Diet Quality

#### 3.3.1. Dietary Guidelines

The mean total dietary guideline scores over 12 were higher for the high SES group (6.48 ± 1.86) compared to the low SES group (5.87 ± 1.95), and the difference in dietary guidelines scores for low and high SES was significant (*P* = 0.0240).

#### 3.3.2. Comparison of the Consumption of Food Items by Low and High Socioeconomic Groups

Consumption of vegetables, refined cereals, full-cream milk and dairy products, low fat protein sources, and sweetened and fatty foods were higher in the low SES group. Fruits, wholegrain cereals, low-fat milk and dairy products, and high fat protein sources were mostly consumed by the high SES group. Differences were significant only for vegetables, wholegrain cereals, refined cereals, and low-fat milk and dairy products (Table 3).

#### 3.3.3. Food Consumption, Calorie-Density, and Cost of Different Food Items

Both fruits and vegetables provide calories at a very high price, with vegetables the most. Refined cereals, full-cream milk and dairy products, and high fat protein sources provide calories at cheaper prices compared to wholegrain cereals, low-fat milk and dairy products, and low-fat protein sources, respectively (Table 4).

When cost per weight was considered, wholegrain cereals (Rs 6.47/100 g) cost almost twice more than refined cereals (Rs 3.50/100 g), and low fat protein sources (Rs 10.50/100 g) were cheaper than high fat ones (Rs 16.06/100 g). Cost per weight of vegetables (Rs 13.09/100 g) was five times less than its cost per calorie (Rs 72.94/100 Cal). Fatty foods and sweetened foods cost more per weight than fruits and vegetables (Table 4).

### 3.4. Socioeconomic Status, Physical Activity Level, and Body Mass Index

A standard multiple regression analysis was conducted, and it was found that SES (17.8%, *P* < 0.05) was the best predictor of BMI followed by physical activity (5%, *P* < 0.05) and dietary habits (3%, *P* < 0.05).

#### 3.4.1. Total Physical Activity Level

As shown in Table 5, low physical activity (PA) was more common in the low SES group (14.9%) as compared to the high SES group (9.20%), but chi-square test revealed that this difference is statistically insignificant (*P* = 0.0970). A very small, negative correlation existed between PA and BMI (*r* = −0.0440, *n* = 200), but which was insignificant (*P* = 0.562).

#### 3.4.2. Time Spent in Physical Activity at School

Participants of high SES practised physical activity at school for a significantly longer time period (207 ± 60.8) than their low SES counterparts (57.2 ± 60.8). There was a small negative correlation between physical activity at school and BMI, (*r* = −0.167, *n* = 191), which was significant (*P* = 0.000) (Table 6).

## 4. Discussion

Given the importance of socioeconomic status (SES) in influencing body mass index (BMI), with diet quality and physical activity (PA) as possible mediators in the link between these two variables, this study yields pertinent results that could be used to address weight problems among Mauritian adolescents.

### 4.1. Socioeconomic Status and Body Mass Index

A significant negative association was obtained between SES and BMI. For instance, participants in the high SES group had lower BMI than those in the low SES group (Table 2). Results reported herein corroborate findings of five studies conducted among adolescents in developed countries like Australia, USA, and Germany [10–12, 14, 15], and studies in Nigeria and Serbia, two developing countries [6, 50]. Documented findings in other developing countries like India and Ghana, however, report a positive association between SES and BMI [16]. A study in Mauritius found no correlation between SES and adolescent BMI [unpublished].

Contradicting results are attributed to two main factors. Firstly, there are no standard methods of categorising SES and each indicator of SES (income, occupation, education) has its own strength and limitations for studying the SES-BMI relationship [50]. For instance, in Iran the number of household members only and neither parental education nor family income correlated positively with BMI [21]. The use of different combinations of SES indicators in above mentioned studies made comparison of findings ambiguous. A review of results on SES and BMI from 333 countries reported that nonsignificant results would be obtained when occupation is used to classify SES [17] and in self-reported surveys, youngsters have found difficulties in describing parental occupation [43]. This possibly explains why the unpublished study in Mauritius, mentioned above, found no correlation between SES and BMI, unlike in the present study. The former used the National Statistics-Socioeconomic Classification, which is based upon parental occupation. In the present study, however, family affluence scale (FAS) [43] was used to classify participant into SES groups. Currie et al. [43] argued that FAS is an equally valid method which integrates several SES indicators in a few questions to permit assessment of family, child, as well as parental SES in a simple way.

Another potential factor which causes variability in the relationships between SES and BMI in different countries is the Human Development Index (HDI). According to McLaren [17], there is an increasing proportion of positive association as one move from countries with high HDI to countries with medium to low HDI. The HDI ranking of Mauritius (66) is below those of Australia (1), USA (6), Germany (10), but above Iran (69), India (100), and Ghana (111) [51]. This might have been a possible contributing factor to explain why unlike Iran, India, and Ghana, the relationship was negative in Mauritius. Interaction between the two factors, namely, a particular country's HDI and choice of SES indicator in studies conducted in that country would make it difficult and complex to compare results concerning SES-BMI relationship between different countries.

A limitation in the current study is that the effect of race (Indo-Mauritian, Afro-Mauritian, Europids) has not been investigated, though in addition to age and sex, it has been found to influence the SES-BMI relationship [52]. Nevertheless, gender was found to have an effect on the relationship between SES and BMI. The negative association between the two variables was significant for females only. However, results are not detailed herein.

### 4.2. Socioeconomic Status and Diet Quality

Our results are also informative for clarifying issues pertaining to SES and diet quality. For instance, it was found that participants of the high SES group adhered to significantly more dietary guidelines than those of the low SES group. To date, there is no available published study pertaining to dietary guidelines and SES. However, there has been other studies reflecting SES and eating habits and similar findings were reported in most of them. Generally, a more healthy diet is consumed by highly educated individuals [13, 22, 23, 25, 53]. A higher level of education will evidently enable parents to better understand dietary requirements and hence encourage healthy eating patterns in their children.

Data from the food frequency table provide robust evidence to support that consumption of vegetables, refined cereals, full-cream milk and dairy products, low-fat protein sources, and sweetened and fatty foods were higher in the low SES group, whereas fruits, wholegrain cereals, low-fat milk and dairy products, and high-fat protein sources were mostly consumed by the high SES group (Table 3). A review which analysed the results of studies from different countries on the consumption pattern of foods according to SES, demonstrated that fresh vegetables and fruits, wholegrain cereals, low-fat dairy products, and low-fat protein sources (lean meat, fish) were more likely to be chosen by groups of high SES. In contrast, the consumption of refined grains, fatty meat, fried foods, and added fats was associated with lower SES [27]. The difference in pattern of food consumption among SES groups has been explained by the fact that healthier and more nutrient-dense foods have higher energy cost [24, 46]. For instance, meat, fruits, and vegetables that offered the highest nutrient density scores were also found to be the most expensive in terms of cost per calorie [28] and may be preferentially selected by higher SES groups. The differences between results reported herein and those on food consumption previously documented [27] may be largely explained by the findings of our market survey. As mentioned, other studies have postulated that consumption of nutrient-dense foods such as vegetables, low-fat milk and dairy products, low-fat protein sources are more prevalent in the higher SES groups because their calories cost more [22, 24, 28]. Our study, however, found that in addition to cost per calorie, the price per weight of food items might also be important in determining food choice among low SES individuals. For example, the market survey revealed that even if vegetables are poor in energy, they were still mostly consumed by the low SES groups, unlike what is reported in other studies [27]. This is because their cost per weight was less than five times their cost per calorie (Table 4). The price per weight of refined cereals was half that of wholegrain cereals, justifying their preference by low SES participants. Similarly, as high fat protein sources cost more (per weight), they were less consumed by the low SES participants, who preferred low fat protein sources (pulses, fish, poultry). The difference in consumption of low fat protein sources was statistically insignificant because they were also the preferred choice of high SES individuals as documented by Darmon and Drewnowski [27]. Even though sweetened and fatty foods cost more per weight, they were mostly consumed by the low SES participants. Ethnicity was not assessed in this study to explain taste, food preferences, and cooking methods [54], which could determine fat and sugar intake. Nevertheless, consumption of sweetened and fatty foods definitely contributes to high calorie intake which places low SES adolescents at a higher risk of overweight and obesity.

### 4.3. Socioeconomic Status, Physical Activity, and Body Mass Index

There have been debates as to whether physical activity (PA) mediates the relationship between SES and BMI among adolescents. Current results have demonstrated that participants in the high SES group were more physically active than those in the low SES group (Table 5). However, this difference was statistically insignificant. Secondly, a negative correlation was found between PA and BMI, but was small and insignificant. These findings suggest that PA is a weak mediator of the SES-BMI relationship. Several previous studies have reported that sedentary behaviour, as defined by time spent watching television or playing video games, as compared to PA is a more potent mediator between SES and BMI [12, 35]. The main reason cited was the characteristics of low SES neighbourhoods which are often described as unsafe and having less recreational resources compared to high SES areas [36]. On the other hand, studies which have found that PA is associated with high SES and influences BMI in this group [31, 34] have used parental education level as SES indicator. They have postulated that educated parents would have more positive value for PA during leisure time. In addition, use of pedometers and accelerometers to measure PA, instead of self-reporting methods, has identified PA as a mediator of the SES-BMI relationship [30]. It can therefore be inferred that using different SES indicators and diverse methods to measure PA by various studies makes comparison of findings complicated [55].

 An important contribution of this present work is that time spent in physical activity at school, a factor previously uninvestigated but which can influence BMI, was significantly higher among the high SES adolescents (Table 6). A significant negative correlation also existed between time spent in physical activity at school and BMI. In the present study, fee-paying private schools were sources of high SES participants. The curriculum of these schools is therefore more favourable for the practice of physical activity compared to private-aided schools which were sources of low SES adolescents. It has previously been reported that high SES schools have more funds to provide infrastructure and equipment for sports and hence more conducive for practising physical activity [12].

Main implications of this study are that overweight and obesity are not related to affluence among Mauritian adolescents. Having a low SES could be a risk factor for pediatric obesity especially in girls in Mauritius. High SES adolescents are more likely to consume a healthy diet than those of the low SES group. Preferences for refined cereals, full-cream milk, fatty and sweetened foods which promote weight gain and might have contributed to higher BMI in the low SES group. Findings support that both cost per calorie and cost per weight may influence food choice of low-income individuals. Physical activity at school, compared to physical activity in general, may better explain the discrepancies in BMI between the two SES groups. This study highlights the importance of effective school nutritional and physical activity intervention programmes, to address overweight and obesity problems among Mauritian adolescents. In particular, special attention should be directed towards the private-aided schools located in rural areas and having mostly low SES students. There have been controversies pertaining to methods used to categorise SES. In future, it would be more appropriate to devise a standard method for this purpose to facilitate comparison of findings between studies. Further research is warranted to examine the effect of cost per weight of food on food selection and verify whether differences exist in time spent in PA at school between low SES and high SES adolescents in other nations. The distance between school and home, and the transportation mean used by children could be investigated as well.

## Figures and Tables

**Table 1:** Classification of food groups according to dietary guidelines.

Food groups	Standard dietary guidelines used	Dietary guidelines categories	Consumption frequencies	Scores
(1) Cooked vegetables	(1) USDA* MyPyramid [39]: “Vary your veggies” (2) AHA** [40]: “Eat fruits and vegetables daily, limit juice intake” (3) MOHQL*** [41]: “Eat more fruits and vegetables”	1	More than once daily	4
			Once daily	3
		0	Once or more per week	2
			Once or more per month	1
			Rarely/never	0

(2) Raw vegetables	Same as for cooked vegetables	1	More than once daily	4
			Once daily	3
		0	Once or more per week	2
			Once or more per month	1
			Rarely/never	0

(3) Fruits	(1) USDA MyPyramid: “Focus on fruits” (2) AHA: “Eat fruits and vegetables daily, limit juice intake” (3) MOHQL: “Eat more fruits and vegetables”	1	More than once daily	4
		0	Once daily	3
			Once or more per week	2
			Once or more per month	1
			Rarely/never	0

(4) Wholegrain cereals	(1) USDA MyPyramid: “Make half your grains whole” (2) AHA: “Eat whole grain breads and cereals rather than refined grain products”	1	More than once daily	4
			Once daily	3
			Once or more per week	2
		0	Once or more per month	1
			Rarely/never	0

(5) Refined cereals	Same as for wholegrain cereals	1	Rarely/never	0
			Once or more per month	1
			Once or more per week	2
			Once daily	3
		0	More than once daily	4

(6) Low-fat milk and dairy products	(1) USDA MyPyramid: “Get your calcium-rich foods. Make sure your milk, yogurt, or cheese is lowfat or fat-free” (2) AHA: “Use non-fat (skim) or lowfat milk and dairy products daily”	1	More than once daily	4
		0	Once daily	3
			Once or more per week	2
			Once or more per month	1
			Rarely/never	0

(7) Full-cream milk and dairy products	Same as for low fat milk and dairy products	1	Rarely/never	0
			Once or more per month	1
		0	Once or more per week	2
			Once daily	3
			More than once daily	4

(8) Low-fat protein sources	(1) USDA MyPyramid: “Go lean with proteins” (2) AHA: “Eat more fish, especially oily fish, broiled or baked”	1	More than once daily	4
			Once daily	3
			Once or more per week	2
		0	Once or more per month	1
			Rarely/never	0

(9) High-fat protein sources	Same as for low fat protein sources	1	Rarely/never	0
			Once or more per month	1
			Once or more per week	2
		0	Once daily	3
			More than once daily	4

(10) Sweetened foods	(1) USDA MyPyramid: “Reduce intake of sugar-sweetened foods and beverages”	1	Rarely/never	0
			Once or more per month	1
		0	Once or more per week	2
			Once daily	3
			More than once daily	4

(11) Fats	(1) MOHQL: “eat foods low in fats especially saturated fats”	1	Rarely/never	0
			Once or more per month	1
			Once or more per week	2
			Once daily	3
		0	More than once daily	4

(12) Oily foods	Same as for fats	1	Rarely/never	0
			Once or more per month	1
		0	Once or more per week	2
			Once daily	3
			More than once daily	4

*USDA: United States Department of Agriculture; **AHA: American Heart Association; ***MOHQL: Ministry of Health and Quality of Life (Mauritius).

**Table 2:** Body mass index for each socioeconomic group.

SES classification	Mean BMI (±SD)	BMI classification (%)			
		Underweight	Healthy weight	Overweight	Obese
Low SES	19.9 (±4.41)	16.7	58.8	14.7	9.8
High SES	18.5 (±2.83)	11.2	76.5	9.2	3.1

**Table 3:** Consumption frequencies of different food items by low and high socioeconomic groups.

Food items	Mean (±SD*)	Results of independent samples *t*-test		
		*P *value	Mean difference	95% CI**
Fruits and vegetables				
Low	2.88 ± 0.686	0.0380	0.215	0.0120 to 0.417
High	2.67 ± 0.753			
Raw vegetables				
Low	2.68 ± 0.946	0.005	0.398	0.119 to 0.677
High	2.28 ± 1.05			
Fruits				
Low	2.89 ± 1.06	0.377	−0.130	−0.419 to 0.159
High	3.02 ± 0.995			
Wholegrain cereals				
Low	0.74 ± 1.19	0.000	−1.82	−2.17 to −1.47
High	2.56 ± 1.31			
Refined cereals				
Low	0.41 ± 0.619	0.000	−0.869	−1.11 to −0.625
High	1.28 ± 1.05			
Milk and DP				
Low	2.08 ± 0.834	0.000	−0.688	−0.926 to −0.451
High	2.77 ± 0.847			
Low-fat milk and DP				
Low	1.47 ± 1.55	0.000	−1.52	−1.90 to −1.15
High	2.99 ± 1.09			
Full-cream milk and DP				
Low	1.29 ± 1.39	0.435	−0.150	−0.529 to 0.229
High	1.44 ± 1.30			
Low-fat PS				
Low	2.63 ± 0.994	0.548	0.0810	−0.185 to 0.347
High	2.55 ± 0.902			
High-fat PS				
Low	1.93 ± 1.12	0.186	−0.195	−0.485 to 0.095
High	2.13 ± 0.925			
Sweetened and oily foods				
Low	2.37 ± 0.766	0.142	0.157	−0.0540 to 0.367
High	2.21 ± 0.721			

*SD: standard deviation; **CI: confidence interval; DP: dairy products; PS: protein sources.

**Table 4:** Cost of food items per 100 Cal and per 100 g.

Food items	Cost (Rs*)/100 Cal	Cost (Rs)/100 g
Fruits	32.48	10.48
Vegetables	72.94	13.09
Wholegrain cereals	1.88	6.47
Refined cereals	0.99	3.50
Low-fat milk and dairy products	13.50	22.17
Full-cream milk and dairy products	8.92	21.91
Low-fat protein sources	9.07	10.50
High-fat protein sources	7.21	16.06
Fatty foods	9.44	24.19
Sweetened foods	5.84	18.76

*Rs: Mauritian rupees; US$ 1 approximately Rs 30.

**Table 5:** Physical activity level of low and high socioeconomic groups.

Physical activity level	SES group				Results of *χ* ^2^ test		
	Low		High		*χ* ^2^ value	*P* value	phi
	*n*	*%*	*n*	*%*			
Low	13	14.9	8	9.20	4.63	0.0970	0.164
Moderate	32	36.8	23	26.4			
High	42	48.3	56	64.4			

**Table 6:** Time spent in physical activities at school by low and high socioeconomic groups.

SES group	Mean ± SD physical activity at school (minutes/week)	Results of independent-samples *t*-test		
		*P *value	Mean difference	95% CI
Low	57.2 ± 60.8	0.000	−151	−177 to −125
High	208 ± 109			
